# Supplementary material for: Prevalence of childfree adults before and after Dobbs v Jackson in Michigan (USA)
Source: PLoS One. 2024 Jan 10;19(1):e0294459. doi: 10.1371/journal.pone.0294459 (PMC10781188; doi:10.1371/journal.pone.0294459)
Supplement: Attachment — Submitted filename: Revision memo.pdf [file pone.0294459.s001.pdf]

Summary of revisions to:
Prevalence of childfree adults before and after Dobbs v Jackson in Michigan (USA)
(PONE-D-23-23066)

Dear Dr. Cioffi and anonymous reviewers,

Thank you for your very helpful and constructive comments, and for the opportunity to revise and resubmit our paper. We have revised our manuscript to address your concerns, and as a result, we believe the new version of the manuscript is improved. In this letter, we include point-by-point responses to the reviewer's comments.

1 Reviewer #1

1.1 "Overall, the paper is well-written, clear, and addresses and important and timely issue."

We are happy to hear you found the paper to be well-written. We agree that this is an important and pressing issue, and we look forward to seeing it in print. We appreciate your very helpful comments, which we think have clarified our findings.

1.2 "The authors need to add an analysis sub-section to the methods section. This sub-section should detail the methods that are used and clarify the comparison categories. For example, in the logistic regression models, the comparison category for childfree adults is not specified. I would assume that it is all other categories of non-parents, but this needs to be explained up front. Also, why use a logistic regression model rather than a multinomial logit model that can show differences across all the non-parent categories? This choice also needs to be explained and justified."

We have added an "Analysis" subsection to the Methods section. Here, we report how we handle missingness, how we pool data, how we evaluate each research question, and where the replication data and code are located.

Research question 2 is narrowly focused on identifying factors associated with whether or not a Michigan adult is childfree. We are primarily interested in whether being childfree is associated with time (i.e., pre- or post-*Dobbs*), but we also control for demographic characteristics. Because this is a binary outcome variable, a logistic regression is most appropriate.

1.3 "In the discussion, there is a bit of slippage in the language. Given that the authors are not using panel data (which is noted later in that section), the language needs to be clear in terms of comparing non-parents before and after Dobbs, rather than looking at interpersonal change over time."

In the revision, we have carefully reviewed the text to ensure that we are clear these are cross-sectional data and all findings apply at the population-level, but do not provide information about possible changes within-person, which would require panel data.

1.4 “Along these lines, I find the sentence on page 7, line 268-269 a bit misleading. Additionally, the comparison category is never noted in the discussion of the results. In the sentence above, it needs to be clarified that this group (childfree individuals) is being compared to other non-parents, some of whom also do not want children, others are ambivalent, etc. The nuance is important for accurately portraying the results.”

This sentence has been revised to more closely match our research question. We have also clarified in the analysis plan that “we estimate a logistic regression predicting whether an adult is childfree,” and therefore the estimates compare childfree individuals to individuals who are not childfree.

In our original submission, our test of research question 1 focused on the full sample, including parents (i.e. the comparison group was all non-childfree adults). In contrast, our test of research question 1 focused on only the non-parent sample (i.e. the comparison group was only non-childfree non-parents). In retrospect, this change in samples and comparison groups was confusing, and your comment helped us identify this. In the revised submission, we have modified our test of research question 2 to also focus on the full sample, including parents. This ensures that the two tests are performed on the same sample and use the same comparison group. It does not change the findings or conclusions, but we believe it does make the analysis clearer and the findings easier to compare across research questions.

1.5 “Relatedly, I would like to see more discussion of the other categories of non-parents. They are described in the beginning of the background section, but rarely mentioned after that. This shift in the text from discussing the categories to zeroing in on the childfree respondents is abrupt and unexplained.”

Thanks for pointing out this disconnect in the background section of our original paper. Our research questions focus on understanding changes in the prevalence of childfree adults in Michigan before and after the *Dobbs* decision. Therefore we have changed the title of our paper to more accurately reflect our research questions and have also tightened the introduction and background sections to focus more directly on childfree adults and their prevalence. We make the case that the population of childfree adults is growing and is large and that it is important to understand societal factors that might be related to its prevalence such as access to reproductive health care. In the section titled “Childfree adults in the US”, for context we briefly describe how childfree adults are uniquely distinct from other types of non-parents. Prevalence rates for other types of non-parents are also included in our analysis for Research Question 1 solely as a point of comparison for understanding changes in the prevalence of childfree adults.

1.6 Please clarify which prevalence estimates you are referring to on page 2, lines 61 and 63. Is it all non-parents or those who are categorized as childfree?”

In the section titled “Childfree adults in the US”, we review several prevalence estimates of childfree adults. For each of the estimates provided, we have been careful to specify that the prevalence rates reflect individuals who are childfree.

1.7 “Why would non-parents be more likely to be categorized as childfree after abortion was no longer federally protected? This is postulated throughout the paper in several places (pg. 3, line 80-81; pg. 5 line 153-154; pg. 7, lines 277-281), but no explanations are offered. I understand that you are not investigating that question in this paper, but offering some possible explanations for why this might be the case is important for justifying the hypotheses.”

Thank you for pushing us to add more detail about why the *Dobbs* decision may be associated with increases in the prevalence of childfree adults. We now discuss this in a couple places throughout the background, providing the most extended discussion at the end of the “Childfree adults in the US” section: “Recent political and legal changes regarding reproductive health care may represent an additional reason that both women and men choose to be childfree. For women, restrictions on access to reproductive health care represent a limit on medical autonomy, and can introduce health risks by blocking access to medically necessary procedures such as abortions for non-viable pregnancies. Although men’s health may not be directly impacted, such restrictions may mean increased health risks to their partners, leading them to re-evaluate their own intentions to have children. Moreover, for both women and men, politically-motivated restrictions on reproductive rights may signal worrying trends toward authoritarianism, leading to concerns about what the future could hold for any children they might have.”

1.8 “In the discussion, it is mentioned that use of permanent methods of contraception have increased in the post-Dobbs period (pg. 8, line 328-9) and it is suggested that this supports the study’s results; however, increases in permanent contraception do not necessarily indicate changes in fertility intentions. Rather, these increases indicate changes in the use of these methods. That is, fertility intentions may have stayed the same, but childfree individuals may have sought out these permanent methods more frequently in order to ensure they remain childfree. Given that the paper’s argument rests on the connection between abortion access and fertility intentions, the links between access and intentions deserve more attention.”

In the revised manuscript, we now observe that the observed increase in prevalance of childfree adults “could also help explain the increase in patients’ requests for permanent methods of contraception like vasectomy, bilateral salpingectomy, and tubal ligation.” Here, we intend to observe that the prevalence of childfree adults increased at about the same time that requests for permanent contraception increased, and thus that the two are correlated. We lack the data to evaluate whether they are causally related, that is, whether formerly non-childfree individuals who became childfree then sought permanent contraception.

2 Reviewer #2

2.1 “The paper is very well written and can be accepted as is for publication.”

We are very happy to hear that you found the paper was well written, and that you have recommended it for publication.

2.2 “What is the age group considered for the study?”

We have clarified that “Each wave is collected from a sample of 1000 Michigan adults *ranging in age from 18 to 96*, and is weighted to make the sample representative of the Michigan adult population with respect to gender, age, race, and education.”

References
